# Ultrasound imaging improves hormone therapy strategies for induction of ovulation and *in vitro* fertilization in the endangered dusky gopher frog (*Lithobates sevosa*)

**DOI:** 10.1093/conphys/coy020

**Published:** 2018-04-28

**Authors:** Katherine M Graham, Cecilia J Langhorne, Carrie K Vance, Scott T Willard, Andrew J Kouba

**Affiliations:** 1 Department of Biochemistry, Molecular Biology, Entomology and Plant Pathology, Mississippi State University, Mississippi State, MS 39762, USA; 2 Department of Wildlife, Fisheries and Aquaculture, Mississippi State University, Mississippi State, MS 39762, USA

**Keywords:** Amphibian, gopher frog, hormone, *in vitro* fertilization, ovulation, ultrasound

## Abstract

Establishing captive breeding populations of amphibians is an important conservation strategy to safeguard against ongoing declines of wild populations and provide broodstock for reintroduction programs. The endangered dusky gopher frog (DGF) has never naturally reproduced in captivity and requires breeding intervention to sustain the population. Methods for inducing ovulation in female DGFs using hormone therapies have not been evaluated. To address this need, we tested four exogenous hormone treatments to induce ovulation in DGFs (*n* = 11/treatment), including: treatment (A) gonadotropin-releasing hormone agonist (GnRHa); (B) GnRHa with dopamine antagonist metoclopramide hydrochloride; (C) GnRHa and human chorionic gonadotropin (hCG) and (D) GnRHa with hCG following two low hCG priming doses. Treatments B, C and D resulted in a significantly greater (*P* < 0.0125) number of ovulating females compared to the control (no hormone); Treatment A was not different from control. For ovulating females, the number of eggs, relative fecundity and cleavage rates of eggs were compared between the four hormone treatments and initial ultrasound grade. Between treatments, there was no difference in number of eggs or relative fecundity; however, Treatments A and D resulted in higher (*P* < 0.05) cleavage rates than Treatment C, but were not different from Treatment B. Ultrasound imaging was used to assess the ovarian state of DGF females prior to and following hormone therapy. A grading scale (Grades 1–5) was developed to characterize ovarian states. Ultrasound grade was found to be a significant (*P* = 0.002) predictor for ovulation following hormone treatment, with only high-grade females (Grades 3–4) ovulating in response to hormones. Ultrasound grade did not influence egg numbers or cleavage rate (*P* > 0.05). Results demonstrate multiple hormone therapies are available for stimulating ovulation in female DGFs and ultrasonography is a valuable tool to inform hormone therapy. Ultimately, these reproductive technologies are critical to enhance breeding and reintroduction efforts for the DGF.

## Teaser

Hormone therapies were tested for efficacy to induce ovulation in the dusky gopher frog. The number of eggs produced, and cleavage rates following IVF were compared between treatments. Ultrasound was explored as a complementary tool to hormone therapy. Findings are critical to enhancing conservation and broodstock production for reintroduction programs.

Editor: Steven Cooke

## Introduction

As amphibian populations have continued to decline globally at alarming rates, (Stuart *et al.*, 2004; IUCN, 2008), the establishment of captive assurance colonies has become a necessary strategy to prevent extinction. A goal of assurance colonies is to breed declining species in captivity for reintroduction and recovery programs (Gascon *et al.*, 2007; Zippel *et al.*, 2011). However, many amphibian species do not breed well, or at all, in captive settings, threatening the long-term sustainability of these programs. Assisted reproductive technology (ART) strategies, including exogenous hormone therapies, *in vitro* fertilization (IVF) and cryopreservation of gametes, can be used to override some of the difficulties breeding and maintaining captive amphibians (Clulow *et al.*, 1999, 2014; Kouba *et al.*, 2012). The small size, high reproductive output and external fertilization of many amphibians, make them well-suited for ART (Roth and Obringer, 2003; Griffiths and Pavajeau, 2008; Clulow *et al.*, 2014); however, because of the diverse reproductive biology of amphibians, there is not a standard therapy that can be applied to all species, and even ART strategies among closely related species can vary widely (Kouba *et al.*, 2012). Given this challenge, it is crucial to explore new ART strategies and tools as well as continue to evaluate and refine existing protocols to ensure the greatest success of captive breeding efforts.

In particular, obtaining eggs is frequently the rate-limiting step for amphibian ART (Kouba and Vance, 2009). A variety of exogenous hormone treatments have been studied to induce ovulation and oviposition in anurans, with treatments targeting different areas of the hypothalamic–pituitary–gonadal axis to stimulate either luteinizing hormone (LH) release from the pituitary, or LH receptors on the gonads (Kouba *et al.*, 2012; Clulow *et al.*, 2014). Gonadotropin-releasing hormone agonists (GnRHa) are widely used in amphibians; however, the necessary doses and response rates vary (Waggener and Carroll, 1998; Whitaker, 2001; Michael *et al.*, 2004; Byrne and Silla, 2010; Clulow *et al.*, 2014). Human chorionic gonadotropin (hCG) also successfully induces ovulation and oviposition in many amphibians (Hollinger and Corton, 1980; Johnson *et al.*, 2002; Michael *et al.*, 2004; Browne *et al.*, 2006a; Lynch *et al.*, 2006; Kouba *et al.*, 2009; Clulow *et al.*, 2012). It is thought to stimulate the gonadal LH receptors (Kouba et al., 2009, 2012; Clulow *et al.*, 2014), although hCG must typically be administered at relatively high concentrations (500–1000 IU), due to the low specificity of hCG by the amphibian receptors (Subcommittee on Amphibian Standards, 1996; Kouba and Vance, 2009). A combination of hormones, such as GnRHa with hCG or pregnant mare serum gonadotropin (PMSG) may have greater efficacy in some species, particularly when low doses of hormones are used to ‘prime’ the oocytes first to assist maturation (Browne *et al.*, 2006b; Kouba et al., 2009; Silla, 2011; Clulow *et al.*, 2012, 2014; Calatayud *et al.*, 2015). Pairing GnRHa with dopamine antagonists is a relatively new approach in amphibian ART. Dopaminergic inhibition of LH has been documented in a number of fish (Dufour *et al.*, 2005; Sokolowska et al., 1985; Peter *et al.*, 1986; Trudeau, 1997; Dufour *et al.*, 2005), and early experiments with *Rana temporaria*, which used hypothalamic lesions and dopamine antagonists to advance spawning and ovulation, demonstrated that dopamine may control LH release in amphibians as well (Sotowska-Brochocka, 1988; Sotowska-Brochocka and Licht, 1992; Sotowska-Brochocka *et al.*, 1994). More recently, studies in northern leopard frogs (*Lithobates pipiens*) have shown promise for the use of dopamine antagonists paired with GnRHa (termed the ‘Amphiplex’ method) to stimulate breeding and ovulation (Trudeau *et al.*, 2013, 2010; Vu and Trudeau, 2016). The technique has also been successfully applied in small-scale trials to several other amphibian species (Trudeau *et al.*, 2010).

A species that exemplifies the challenges of captive amphibian breeding, and is at high-risk of extinction without intervention, is the dusky gopher frog (DGF; *Lithobates sevosa*; also known as the Mississippi gopher frog). Once found throughout the southeastern USA, only two small populations of ~100 adult DGFs remain in Mississippi due to habitat loss and urbanization (Hammerson *et al.*, 2004; USFWS, 2012). To protect this critically endangered species until habitat could be restored, a number of individuals were taken into zoos as a hedge against extinction and loss of genetic diversity. However, the DGF has never reproduced naturally in captivity, and to date, studies investigating ART strategies for the DGF are limited and primarily focused on males. Protocols for hormonal induction of spermiation and cryopreservation of spermic urine have been developed (Germano *et al.*, 2011; Langhorne *et al.*, 2013), but methods to reliably induce ovulation and oviposition in DGF females remain lacking. Preliminary trials by our research group to induce ovulation tested two low priming doses of hCG administered several days apart, followed by a higher dose of hCG paired with GnRHa (Kouba *et al.*, 2011). Initially, this ovulation protocol was unsuccessful; however, when treated with the same hormone therapy again several months later, females ovulated and eggs were collected by manual expression (females did not oviposit naturally). While this method ultimately resulted in eggs, it required multiple rounds of hormone treatments and manual intervention to obtain the gametes. Studies are needed to explore modified or new hormone therapies in the DGF, which may stimulate more consistent ovulation and oviposition response. If successful, this would increase efficiency of captive breeding efforts and offspring production for reintroduction programs.

Arguably, one of the greatest challenges for researchers utilizing hormone therapies in amphibians is that the reproductive state of the animals is not readily distinguishable, and changes in ovarian state following a treatment are difficult to ascertain. Ultrasonography may be a valuable tool for use alongside hormone therapy to track changes in follicle and egg development. The skin of amphibians is readily penetrated by ultrasound waves and anesthesia is not necessary, making it an easy and relatively non-invasive assessment tool (Mannion, 2008; Schildger and Triet, 2001; Stetter, 2001). Despite its relative ease of use, ultrasound imaging of amphibians has been primarily limited to medical examinations. Only a few studies of anurans have used ultrasonography for assessment of reproductive status or visualization of follicles and eggs (Johnson *et al.*, 2002; Reyerand Bättig, 2004; Calatayud *et al.*, 2018). We have already implemented ultrasonography in DGFs for sex identification (Graham *et al.*, 2016), and here we expand the use of this technology to provide new insights into the ovarian state and responses of DGFs to hormone therapies, and to aid in determining best candidates for ART.

The objectives of this study were to: (i) evaluate the success of four exogenous hormone treatments to induce ovulation and egg expression in female DGFs; and (ii) validate and utilize ultrasound to assess ovarian state prior to and during hormone treatment. The success of each treatment was measured by the number of females ovulating following hormone therapy (as determined by subsequent manual expression of eggs), and the number and quality of eggs produced. For ultrasound data, a grading scale was developed to aid in consistent categorization of ovarian state and could be used to predict which females were most likely to respond to hormone therapy. This study evaluates novel and refined hormone therapies to improve assisted reproduction in DGF females and offers evidence for the value of ultrasound as a complementary tool when used alongside hormone therapies. These outcomes are critical for the long-term sustainability of the DGF captive assurance population, and for increasing offspring production for reintroduction programs.

## Materials and methods

### Animals

A total of 26 sexually mature female DGFs housed at Mississippi State University’s (MSU) Amphibian Conservation Lab were used in the study. All animals were captive bred and reared at the Memphis Zoo or Omaha’s Henry Doorly Zoo and Aquarium, and transferred to MSU prior to the study. Females ranged in age from 4 to 6 years old. Body weights ranged from 29.7 to 67.7 g, and snout-vent length (SVL) measurements (performed in the month prior to hormone treatments) as described in Graham *et al.* (2016) ranged from 56.4 to 74.9 mm. Females were implanted with passive internal transponder tags for identification.

All husbandry practices and treatment protocols were approved by MSU’s Institutional Animal Care and Use Committee (IACUC #10–082). Frogs were housed in plastic polycarbonate tanks (46 × 66 × 30 cm; Habitat Systems Limited, Des Moines, IA) under local natural light conditions, with one to four conspecifics, in both single and mixed-sex housing arrangements. Tanks were supplied with moistened organic moss substrate, plastic hides and aged tap water. During treatment periods (10 days), females were transferred to individual holding tubs (19 × 29.5 × 15.5 cm) to monitor study parameters. Dampened moss and aged tap water were provided in each holding tub. Animals were checked once per day during which all study parameters were measured to minimize handling stress. In both settings, frogs were provided prey items (crickets, mealworms and wax worms) three times per week. Worms were gut loaded prior to feeding using Repashy ‘Superload’ supplement (Repashy Ventures Inc., Oceanside, CA). Crickets were gut loaded with fresh fruits and vegetables sprinkled with Repashy diet, and crickets were dusted with calcium (Fluker’s Calcium with D_3_; Fluker Farms, Port Allen, LA) before distribution.

### Ultrasound validation and ovary imaging

Transabdominal ultrasonography was used to monitor the state of ovarian follicles and eggs, prior to and during hormone treatments. We hypothesized that: (i) females may show differences in ultrasound patterns based on ovarian state; (ii) the ovarian state of a female prior to treatment may affect her response to the hormone therapy and (iii) females may demonstrate changes in their ultrasound profiles following hormone treatment. Ultrasound imaging was performed using a Sonosite MicroMaxx ultrasound (Sonosite Inc., Bothell, WA) equipped with a 38-mm broadband linear array transducer (range 13–6 MHz), or a Sonosite Titan ultrasound with 38-mm broadband linear array transducer (range 10–5 MHz) set to a scan depth of 2.7 cm on the breast/tissue setting. The female’s abdomen was wetted with aged tap water to act as a conductor, and the transducer was gently held against the abdomen to obtain an image of the ovaries.

**Figure 1:**
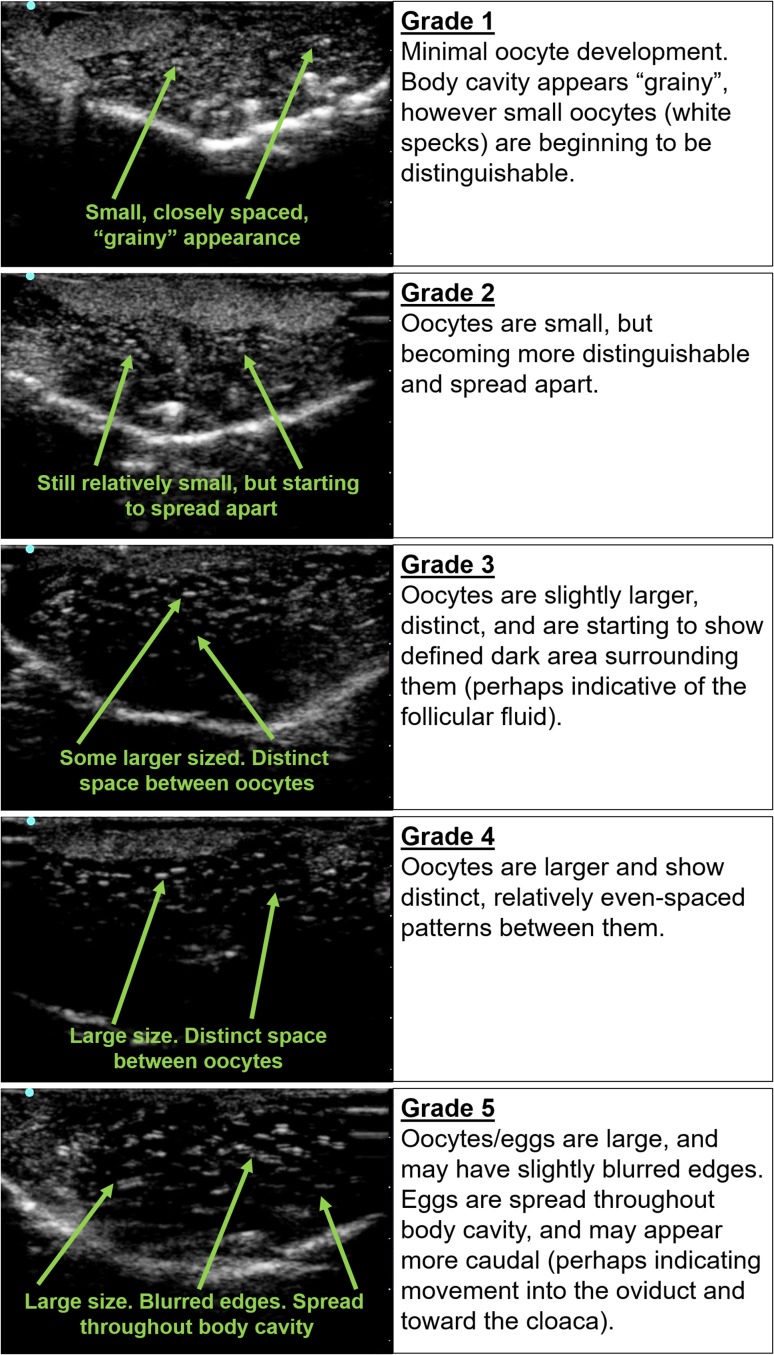
Transabdominal ultrasound grading scale (Grades 1–5) for dusky gopher frogs.

To consistently describe the ovarian state across imaging, an ultrasound grading scale was developed, which categorized the imaged follicles and eggs on a scale from 1 to 5 based on the hyperechoic and hypoechoic (light and dark) patterns of the ultrasound image. A female of Ultrasound Grade 1 was considered to have low follicular development, while a female of Grade 5 had well-developed eggs ready for expression. Images and descriptions for each grade are shown in Fig. 1. The patterns seen in Grades 1–4 ovaries were representative of follicle/oocyte complexes and are referred to as ‘oocytes’. The differences in the Grade 5 images were shown to be the result of ovulation of mature eggs; thus, the gametes in these images are referred to as ‘eggs’. The term ‘egg’ is also used for gametes that were oviposited by or manually expressed from a female.

Due to limited use of ultrasound to classify ovarian state in anurans, we conducted an experiment to validate that ultrasound grades were distinct and could be used to categorize differences in ovarian state. Given that comparison of ovaries required sacrifice of the animal, and considering the endangered status of the species, this comparison study was limited to one female of each ultrasound grade. Females selected were from a genetically redundant research population, and approval for euthanasia was included within the IACUC. Frogs were humanely euthanized in a buffered 5 g/l solution of MS-222 according to recommended procedures (Torreilles *et al.*, 2009; Pessier and Mendelson, 2010). Ovaries were removed from each female and examined under an Omano 3344 stereo-microscope. Upon examination, five types of oocytes were distinguishable based on size and coloration: small, translucent; small, white; small, undergoing pigment accumulation; large, undergoing pigment accumulation; and large, dark, with clear animal and vegetal poles. ImageJ (1.49v, National Institutes of Health, USA) was used to measure the diameter length (mm) of each type of oocyte, with an average length calculated based on measurements from 30 oocytes of each type. To evaluate differences between ultrasound grades, a total of 100 oocytes were counted from randomly selected portions of the ovaries for each female, and the percentage of each oocyte type present on the ovary was calculated for each grade.

### Hormone treatments

Four hormone treatments were tested during this study for efficacy at inducing ovulation in DGFs. Ovulation was concluded based on successful manual expression of eggs from a female (the term ‘ovulation’ will be used throughout the paper to describe ovulation of eggs from the ovary and the subsequent collection of the eggs from a female by manual expression). Treatment trials were performed at two different times of the year, early spring and late summer-fall. The DGF is generally described as a spring breeder; however, breeding is also observed in the fall in response to hurricane rains (Richter et al., 2003; Hammerson *et al.*, 2004). Trial 1 was performed from August through early December 2014 (Fall 2014), and Trial 2 was performed from March to May 2015 (Spring 2015).

Hormone treatments, doses and timelines are outlined in Table 1. Treatments included: [A] a synthetic GnRH agonist (GnRHa; #L4513, Sigma Aldrich, St. Louis, MO); [B] GnRHa paired with dopamine receptor antagonist metoclopramide hydrochloride (MET; #M0763 Sigma Aldrich); [C] GnRHa paired with hCG (#C1063, Sigma Aldrich) and [D] two low priming doses of hCG followed by a higher dose of GnRHa paired with hCG. The day of the ovulatory dose was called Day 0 of the trial; priming doses were given on Day −4 and Day −1 to the ovulatory dose. Females were weighed just prior to treatment administration and all hormone doses were prepared on a per gram body-weight basis (μg/g or IU/g body weight). Stock solutions of hormones were prepared for use as follows: GnRHa lyophilized powder was reconstituted in distilled water to a concentration of 1 μg/μl, aliquoted, and stored at −20°C prior to use, as the hormone degrades quickly at room temperature. Powdered MET was diluted in 0.7% saline solution to a stock concentration of 5 μg/μl, as previously published (Trudeau *et al.*, 2010). Fresh MET stock was prepared for each frog prior to administration. Vials of 2500 IU hCG were reconstituted in 500 μl phosphate buffered saline solution (PBS; #0928103 MP Biomedicals, Santa Ana, CA), to make stock solutions of 5 IU/μl. The hCG stock was used immediately or stored at 4°C (for <5 days). Individual hormone doses were prepared in sterile tubes (Biopur #022600028, Eppendorf North America, Hauppauge, NY) from the described stock solutions, and administered intraperitoneally in the body cavity space near the gonads using BD PrecisionGlide^TM^ 27G × ½-inch needles (#305 109, BD, Franklin Lakes, NJ) and 1-ml Luer syringes (NORM-JECT #4010-200V0, Henke-Sass Wolf, Tuttlingen, Germany) immediately following preparation.

**Table 1:** Hormone treatment doses and timelines. Doses are presented in μg or IU per gram of body weight (μg/g or IU/g) for an individual. Day 0 refers to the day of the ovulatory dose, while Day −4 and Day −1 refer to the number of days before the ovulatory dose. Treatments were modeled off of Kouba *et al.* (2011) (Treatment C and D) and Trudeau *et al.* (2010) (B). Treatment A was used to test the efficacy of a GnRH agonist alone

	Day of treatment and dosage timeline		
Treatment	Day −4	Day −1	Day 0
A			0.4 μg/g GnRHa
B			0.4 μg/g GnRHa + 10 μg/g MET
C			0.4 μg/g GnRHa + 13.5 IU/g hCG
D	3.7 IU/g hCG	3.7 IU/g hCG	0.4 μg/g GnRHa + 13.5 IU/g hCG

To control for the fact that the ovarian state of a female may affect her response to hormone treatment, we assessed the ovarian state of each female (using the 1–5 grading scale described above) prior to assigning females to a hormone treatment for each trial. Females were grouped by ultrasound grade, then frogs from each grade group were randomly assigned to a treatment, such that there were equal numbers of females of a given ultrasound grade across each of the four hormone treatments (A, B, C and D). Because the frogs were assigned based on their natural grade (no attempts to alter the grades were made), there were different numbers of each ultrasound grade (Grades 1–4) represented within and between each trial (Table 2). Due to the limited number of DGFs in captive breeding populations, the same females were used in both trials. Prior to Trial 2, each female’s ovarian state was re-graded and she was randomly assigned to a different hormone treatment than the one received in Trial 1.

**Table 2:** Number of females of each ultrasound grade assigned to treatments for each season. The ovarian grade was assessed at the start of the trial (before hormone treatment). Although it was not possible for each treatment to have the same number of females across all grades, the number of females within each ultrasound grade was kept consistent within a trial

Treatment	Fall (Trial 1)			Spring (Trial 2)		
	Grades 1–2	Grade 3	Grade 4	Grades 1–2	Grade 3	Grade 4
A	1	1	3	1	3	2
B	1	1	3	1	3	2
C	1	1	3	1	3	2
D	1	1	3	1	3	2

### Monitoring ultrasound grade, ovulation, and egg quantity/quality in hormone treated females

Following the ovulatory hormone dose (Day 0), each female was checked once daily for weight (g), presence of eggs and ultrasound grade for the next 10 days, or until all eggs were expressed. During each check, an attempt to manually express eggs was performed. Previous experience in our lab showed DGF females rarely deposit eggs on their own, and manual expression of eggs is necessary. Manual expression also allowed for IVF procedures to be conducted in a standardized manner. If present, ovulated eggs could be manually expressed by lifting open the female’s cloaca with a small probe while simultaneously providing gentle squeezing pressure to the sides of the abdomen, with pressure directed in a craniocaudal direction. The egg check was performed ‘blind’ (before daily ultrasound), to ensure the ultrasound grade of the female did not influence the amount of squeezing stimulus the frog received. Following the egg check, the ultrasound grade (1–5) was recorded.

If eggs were not expressed by Day 10 post-hormone treatment, Round 1 of treatment was concluded and the frog was returned to its normal tank. Since our ultimate goal was to collect gametes for reproduction and research purposes, females that did not ovulate were treated again with hormone therapy after a rest period. Approximately 1 month following the initial treatment, females underwent ultrasound imaging, were reweighed and reinjected with the same, previously assigned hormone therapy (Round 2 of treatment). The same daily check protocol was used as in Round 1. If the animal failed to ovulate (no eggs) by Day 10 in Round 2, the frog was rested ~1 month, before administration of a third and final hormone treatment (Round 3) for the trial, following the same procedure above. If the animal did not ovulate after the third round of hormone therapy, it was deemed a ‘non-responder’ for the trial, and hormone treatments were discontinued until Trial 2.

We defined that a female ovulated in response to hormone therapy if eggs were manually expressed during any of the daily checks. Females were handled daily for egg expression until all eggs were deposited based on visualization by ultrasound (returned to Grades 1–2). To determine if there was a difference in the amount or quality of eggs between the four hormone treatments, three measures were taken for each ovulating female: eggs number, relative fecundity and average cleavage rate. Egg number consisted of the total number of eggs expressed from an ovulating female. Relative fecundity was calculated as the total number of eggs produced by a female divided by her body weight at injection as described in Trudeau *et al.* (2013). Relative fecundity accounts for the fact that larger females tend to lay more eggs than smaller females and was used to confirm that potential differences in egg numbers were related to hormone treatment and not the size of a female. Lastly, average cleavage rate was used as a proxy for egg quality. Average cleavage rate was determined by performing IVF on a subset of expressed eggs and calculating the mean percentage of eggs that successfully reached 2–4 cell-stage for each female.

*In vitro* fertilization was performed using our lab’s standard protocol on the first day eggs were expressed from a female. Sperm used for IVF was collected fresh (within 48 h) from DGF males in the colony (sperm that was not used immediately was stored cold at 4°C temperatures until use (we have observed DGF sperm samples in these conditions to maintain motility and viability, Graham, unpublished)). Males were injected intraperitoneally with a combination of 500 IU hCG and 15 μg GnRHa (Germano *et al.*, 2011; Kouba *et al.*, 2011). Spermic urine was collected in a clean dish by inserting a small piece of catheter tubing (#BB31785-V/5; Scientific Commodities Inc., Lake Havasu City, AZ) into the male’s cloaca to stimulate urination. Percentage of sperm motility was assessed on 100 sperm prior to use in IVF, and only spermic urine samples with >50% motility were used. Sample concentrations were calculated using a Neubaeur hemocytometer. Since males could only be stimulated to spermiate every few weeks and only a limited volume of spermic urine could be collected (typically ~200–500 μl) from each male, the same males and sperm samples could not be used for all fertilization trials. Therefore, two males were randomly selected for spermiation each time eggs were collected.

Fertilization was performed by expressing ~20–30 eggs into a 150 mm × 15 mm-petri dish (FisherBrand #0875714; Fisher Scientific, Pittsburgh, PA). A rounded edge probe was used to open the cloaca and guide eggs into a clump on the dish. In total, six dishes of eggs were expressed for each female, with three dishes assigned to each of the two males selected for spermiation. Sperm concentration and volumes were standardized across the dishes by diluting sperm in non-spermic frog urine to a concentration of ~1000 sperm per egg (20 000–30 000 sperm per dish) in 100 μl volume. These concentrations and volumes are similar to optimized ranges in other fertilization studies (Hollinger and Corton, 1980; Edwards *et al.*, 2004). Sperm was diluted just prior to use, then pipetted directly onto the eggs, with one 100-μl sperm aliquot used per dish. Eggs were then left for a 5-min ‘dry fertilization’ period to allow the sperm to penetrate the egg jelly. After this period, dishes were flooded with aged tap water and left undisturbed at room temperature to allow development. Cleavage rate (%) was determined ~4–6 h later by counting the number of 2–4 cell embryos in each dish out of the total number of eggs in the dish. The cleavage rate for each ovulating female was calculated by averaging the cleavage rates across all of a female’s dishes. A control was also established for each trial, where eggs were exposed to 100 μl of non-spermic frog urine to confirm parthenogenetic activation or cleavage did not result from mechanical stimulation of the eggs during expression or the IVF process (Rugh, 1962; Toro and Michael, 2004).

### Control treatment

Each female received a control treatment of 200 μl PBS (phosphate buffered saline; no hormone) prior to participation in hormone treatment trials to confirm that exogenous hormone stimulation was necessary for ovulation. Females were then checked daily for 5 days for the presence of eggs (using the procedure outlined above). A shortened trial was used to avoid stress and injury to the animal when attempting to express non-ovulated eggs. Daily ultrasound was also performed pre-, during and post-control to confirm there were no changes in ovarian state. The control treatment was repeated three times (once per month for 3 months), to mirror the same conditions as those used during hormone trials.

### Statistical analysis

Analysis of hormone treatments was conducted in three parts. First, to compare for differences in the number of ovulating females between the control and all hormone treatments, a McNemar’s mid-*p* test was used (Fagerland *et al.*, 2013). Subsequent McNemar’s mid-*p* tests were then performed to compare the number of ovulating females from each individual hormone treatment versus the control. A Bonferroni correction was applied to correct for the multiple comparisons, with the *P*-value for significance adjusted to *P* < 0.0125 (*α* = 0.05/4 treatment groups = 0.0125). Second, for hormone trial data, a binary logistic regression was used to determine which factors may have influenced female ovulation. In the model, hormone treatment (Treatments A, B, C and D), initial ultrasound grade (Grades 1–4), trial (Fall versus Spring) and body weight of a female at injection were explored as predictors for ovulation success. The third analysis, for ovulating females only, two-way ANOVAs were used to compare for differences in egg number, relative fecundity and average cleavage rate of eggs based on the factors of hormone treatment and ultrasound grade. Shapiro–Wilks tests were used to confirm normality; cleavage rate data were arcsine-transformed to meet normality assumptions. Levene’s test was used to confirm homoscedasticity of variances. Post hoc comparisons were made using Tukey HSD. For ovulating females, we also explored if the number of rounds of hormone treatments (1, 2 or 3) required for ovulation was related to ultrasound grade or season using Fisher’s exact tests, or body weight using a Spearman rank-order correlation coefficient. We also used Spearman’s to explore correlations between body weight, SVL, or the rounds of hormone treatments, and the response variables of egg number, relative fecundity or cleavage rate. All descriptive data are expressed as mean ± standard error (SE). Significance for all tests was set at *P* < 0.05. Analyses were conducted using SPSS (Version 23; IBM Analytics; Armonk, NY).

## Results

### Ultrasound validation and ovary imaging

**Figure 2:**
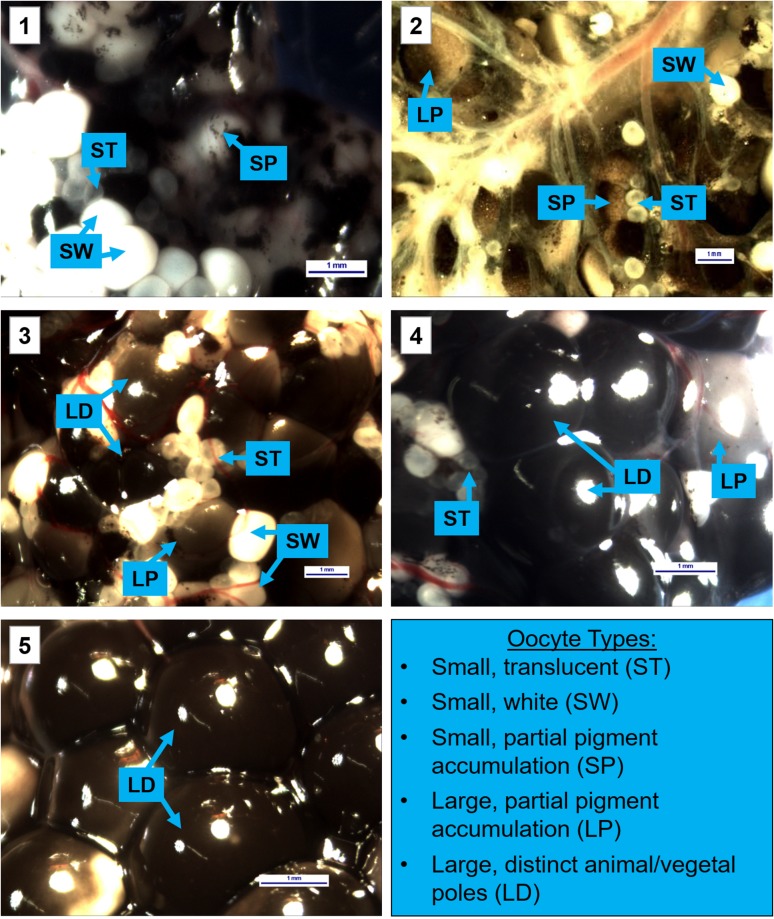
Ovaries from DGF females of each ultrasound grade (Grades 1–5 in numbered panels). There were five main types of oocytes observed on the ovaries; categorized based on size and coloration: small, translucent (ST); small, white (SW); small, undergoing pigment accumulation (SP); large, undergoing pigment accumulation (LP) and large, dark, with clear animal and vegetal poles (LD). The labeled blue boxes and arrows show examples for each type of oocyte. The white box in the lower right corner of each panel contains a blue scale bar of 1 mm relative to the panel. The lower grade ovaries (Grades 1–2) predominately had smaller oocytes, while higher grades (Grades 3–5) showed greater numbers of large oocytes.

Ultrasound imaging was a useful tool for tracking changes in the ovarian state of a female throughout the study. Differences in the hyperechoic (light) and hypoechoic (dark) patterns were used to characterize each grade. An example ultrasound image and description for each grade can be seen in Fig. 1. Direct examination of the ovaries during the validation experiment, showed distinct differences between the five ultrasound grades (Fig. 2). Ovaries from the lower grades (Grades 1 and 2) were dominated by smaller oocytes. For Grade 1, many of the oocytes were translucent or white in color, with some containing small accumulations of pigment. The Grade 2 female tended to have smaller oocytes overall compared with the higher grades, although a small number of oocytes in this female had increased in size with some showing distinct animal (heavily pigmented) and vegetal (lighter colored) poles. Increased pigment accumulation in the small oocytes was noted. For the higher grades (Grades 3, 4 and 5), smaller translucent and white oocytes were still present throughout the ovaries, but larger size oocytes were the dominant feature. The Grade 3 ovaries dominated more body cavity space and had a greater number of large oocytes compared with the lower grades. Many of these larger oocytes also showed distinct animal and vegetal poles. The Grade 4 ovaries had primarily large oocytes, which were heavily pigmented with distinct poles, and were similar in appearance to the mature eggs seen following egg expression, but were still attached to the ovary. The ovaries took up much of the body cavity space, crowded other internal organs, and caused slight protrusion of the female’s abdomen and sides. Finally, the Grade 5 female showed large, mature oocytes on the ovary, similar to Grade 4, but in addition, some of the eggs had been ovulated from the ovary and were present in the oviduct. This was the only grade which presented with ovulated eggs (Table 3). When extracted from the oviduct, these eggs had a slight jelly-like coating. The body cavity of the Grade 5 female was dominated by the enlarged ovaries and eggs, with the other organs crowded, and her abdomen and sides were distended. Table 3 lists the average size (length) of each oocyte type and provides the percentage for each oocyte type on the ovaries for each ultrasound grade.

**Table 3:** Types of oocytes present on DGF ovaries. Column 2 shows mean length (in mm) and standard error (SE) calculations made by measuring 30 oocytes or eggs of each type. The percentages of each oocyte type found on the ovaries of females from each ultrasound grade are listed in columns 3–7 (calculated using a count of 100 oocytes from randomly selected surfaces of the ovary). The last row notes the presence and average size of ovulated eggs in the oviduct

Oocyte description	Length (mm) [mean ± SE]	Grade 1 (%)	Grade 2 (%)	Grade 3 (%)	Grade 4 (%)	Grade 5 (%)
Small, translucent	0.46 ± 0.02	33	24	8	14	13
Small, white	0.93 ± 0.03	29	21	25	15	21
Small, partial pigment accumulation	0.85 ± 0.02	34	25	16	15	10
Large, partial pigment accumulation	2.04 ± 0.04	4	22	28	23	15
Large, distinct animal/vegetal poles	2.15 ± 0.04	0	8	23	33	41
Eggs present in oviducts	2.88 ± 0.05	No	No	No	No	Yes

Following administration of the hormone treatments, some females demonstrated changes in their ultrasound grade. All ovulating females showed an increase to ultrasound Grade 5 prior to egg expression. The ultrasound image of a Grade 5 female looked distinct from the other grades, and the egg mass was frequently observed to move toward the caudal end of the body cavity prior to successful egg expression. Following egg expression, the ovaries reverted to Grade 1 or 2 on ultrasound images. Only females that were a Grade 3 or 4 at the start of a trial ever ovulated following hormone treatments, although not all Grade 3 and Grade 4 females ovulated. No females recorded as Grade 1 or 2 at the start of a trial ovulated following hormone treatment.

### Comparison of ovulation during control and hormone treatments

**Figure 3:**
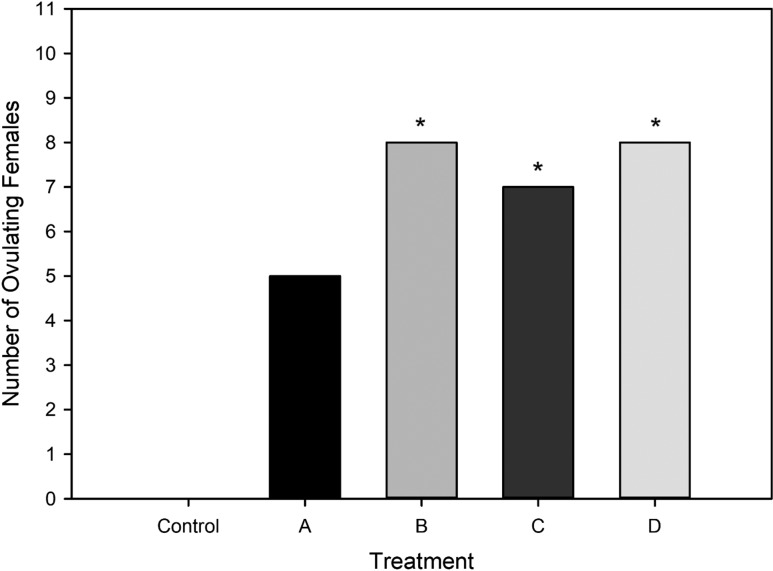
Number of ovulating females per Treatment group. No females deposited eggs during the control (no hormone) trials; (*) indicates a significantly greater number of ovulating females for the hormone treatment compared with control treatment (*P* < 0.0125, Bonferroni corrected value).

No females ovulated in response to the control treatment (PBS). The number of ovulating females was significantly higher (*P* < 0.0001; McNemar’s mid-*p*) following hormone treatment (Fig. 3). There were 28 occurrences of ovulation across all hormone treatments and trials. Of the 11 females in each hormone treatment group, 5 ovulated in response to Treatment A, 7 to Treatment C, and 8 females ovulated following Treatments B and D. Subsequent comparisons using McNemar’s mid-*p* test found the number of ovulating females was significantly different between the control and Treatments B (*P* = 0.004), C (*P* = 0.008) and D (*P* = 0.004). Treatment A was not significant (*P* = 0.032) when the Bonferroni corrected *P*-value of 0.0125 was applied to account for multiple comparisons.

A binary logistic regression was used to evaluate which factor(s) may have influenced ovulation success. In the model, hormone treatment, ultrasound grade, trial and female body weight were used as predictors for the outcome of successful ovulation. This model was significant (*χ*^2^_(4)_ = 28.342, *P* < 0.0001), suggesting the variables could be used to predict ovulation. The model showed a moderately strong relationship between the prediction and outcome, explaining 65.0% of the variance (Nagelkerke *R*^2^) in ovulation success, and correctly predicted ovulation in 84.1% of cases. Ultrasound grade was found to be a significant predictor for ovulation (Wald = 9.289, df = 1, *P* = 0.002; Table 4), with a higher initial ultrasound grade associated with successful ovulation by a female. Ovulation was not predicted by hormone treatment (A, B, C, D), season (fall or spring), or female body weight.

**Table 4:** Binary logistic regression model predictor values. Hormone treatment, ultrasound grade, season and female body weight at injection were used as predictors, with ultrasound grade shown to be a significant (*) factor to predict ovulation in a female

	B	SE	Wald	df	Significance	Exp(B)
Hormone treatment	0.674	0.446	2.283	1	0.131	1.963
Ultrasound grade	2.853	0.942	9.167	1	0.002*	17.331
Season	−2.047	1.165	3.090	1	0.079	0.129
Body weight	−0.004	0.049	0.006	1	0.939	0.996
Constant	−6.611	3.058	4.675	1	0.031	0.001

### Treatment comparison for ovulating females

In total, 16 925 eggs were collected across all hormone treatment groups and trials in this study. For ovulating females, the eggs could first be expressed on Day 2 or Day 3 post-hormone administration. The number of days to complete egg expression ranged from 1 to 6 days. To avoid injury to the animals, females were handled each day for egg expression until eggs were no longer easily exiting the cloaca with mild pressure.

The mean number of eggs per female, relative fecundity and cleavage rate for the four hormone treatments can be found in Table 5. Table 5 also lists the median number of treatment rounds required for each hormone therapy until a female ovulated. There was no association between the number of rounds of hormone treatments a female required before ovulating and her ultrasound grade (*P* = 1.00) or the season (*P* = 0.101; Fisher’s exact test). The number of rounds of hormone treatments was also not correlated with female body weight (*P* = 0.750), SVL (*P* = 0.973), egg number (*P* = 0.490), relative fecundity (*P* = 0.839) or cleavage rate (*P* = 0.116). Body weight and SVL were not correlated with the number of eggs (BW: *P* = 0.320; SVL: *P* = 0.206) or the cleavage rate of eggs (BW: *P* = 0.214; SVL *P* = 0.258).

**Table 5:** Mean number of eggs per female, relative fecundity, and cleavage rate for each hormone treatment. Data are presented as mean ± SE followed by range in parentheses. Different superscript letters within the cleavage rate column denote a significant difference (*P* < 0.05) based on post hoc comparisons (Tukey HSD). Median # trt rounds refers to the median number of rounds of hormone treatments until ovulation occurred

Treatment	# Ovulating females	Median # trt rounds	Number of eggs	Relative fecundity	Cleavage rate (%)
A	5	2.0	685 ± 69 (477–849)	14.6 ± 2.4 (7.0–20.2)	56.9 ± 9.8^a^ (31.0–92.0)
B	8	1.5	625 ± 58 (409–884)	12.7 ± 1.3 (8.6–17.9)	54.7 ± 6.8^a,b^ (29.8–81.8)
C	7	1.0	481 ± 62 (200–672)	12.8 ± 1.9 (3.5–19.4)	28.7 ± 8.2^b^ (3.3–62.8)
D	8	1.0	642 ± 64 (411–898)	14.6 ± 1.2 (9.3–19.7)	59.1 ± 5.7^a^ (38.7–85.1)

**Figure 4:**
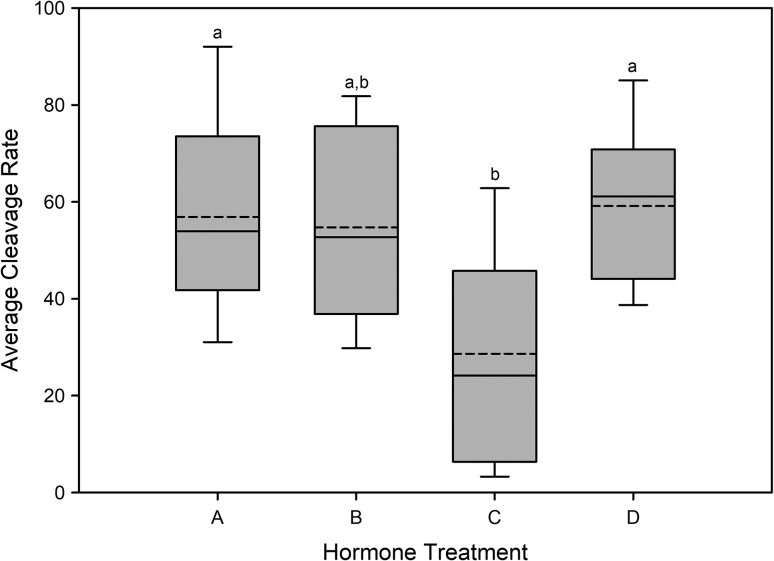
Boxplot comparison of cleavage rates for ovulating females for each treatment. Boxes represent the first and third quartiles, with the median shown by the solid line and the mean indicated by the dashed line. Treatments with differing lowercase letters have significantly different cleavage rates.

There was no significant interaction (*P* > 0.05) between hormone treatment and ultrasound grade for the number of eggs, relative fecundity and cleavage rate (Table 6). The main effect of ultrasound grade was also not significant (*P* > 0.05) for any of the response variables (Table 6). The main effect of hormone treatment was not significantly different for number of eggs or relative fecundity; however, cleavage rate was significantly different (*P* < 0.05) between the treatments (Table 6). Post hoc comparisons showed Treatment C (hCG and GnRHa, with no priming doses) with a mean cleavage rate of 28.7 ± 8.2% was significantly lower (*P* < 0.05) compared with Treatments A and D, but was not significantly different from Treatment B (Fig. 4). Cleavage rates across all groups ranged from 3.5 to 92.0%. There was no evidence of cleavage for the control eggs treated with non-spermic urine.

**Table 6:** Two-way ANOVA results for the response variables: number of eggs per female, relative fecundity and cleavage rate. Factors were set as hormone treatment (Hormone trt) and ultrasound grade (US grade). Cleavage rate was found to be significantly (*) different for the main effect of hormone treatment

Model	Number of eggs	Relative fecundity	Cleavage rate
Hormone trt x US grade	*F*_(3,20)_ = 0.20; *P* = 0.89	*F*_(3,20)_ = 8.07; *P* = 0.76	*F*_(3,20)_ = 0.24; *P* = 0.87
Hormone trt	*F*_(3,20)_ = 1.33; *P* = 0.29	*F*_(3,20)_ = 0.22; *P* = 0.88	*F*_(3,20)_ = 3.39; *P* = 0.04*
US grade	*F*_(1,20)_ = 0.58; *P* = 0.46	*F*_(1,20)_ = 0.53; *P* = 0.47	*F*_(1,20)_ = 2.47; *P* = 0.13

## Discussion

This study demonstrates the success of new and refined hormone therapies for inducing ovulation in the DGF, and highlights ultrasound as a tool to improve reproductive monitoring during hormone therapy. Although all four hormone treatments successfully induced ovulation in the DGF, Treatment A (GnRHa alone) had the lowest number of ovulating females and was not significantly different from the control. Treatment A also required the greatest number of rounds of hormone treatment (median of 2.0) for ovulation induction compared with other treatments. Treatment A relies entirely on activating the animal’s endogenous reproductive axis to produce a sufficient LH surge from the pituitary for ovulation (Clulow *et al.*, 2014), thus the dose tested here may have been too low, or GnRHa on its own may not sufficiently stimulate the pituitary to cause an ovulatory LH release in DGFs. The other treatments tested here pair GnRHa with another compound, which likely explain the higher rates of ovulation observed for these treatments. For females that did not ovulate following the initial round of hormone treatment, the prior rounds may have acted as a priming dose, assisting in the development and maturation of the eggs. However, the additional rounds of hormone treatments were not correlated with greater egg numbers or cleavage rates. Additional treatment trials or performing endocrine analyses concurrently to hormone therapy may clarify if there is a superior hormone treatment for inducing ovulation in the DGF.

Ovulation rate was also not different between the two seasons. As demonstrated in this study, hormone therapy can be used successfully in both spring and fall seasons in the DGF. The DGF is considered a spring breeder, however fall breeding is occasionally observed when heavy storms fill ephemeral breeding ponds (Richter *et al.*, 2003; Hammerson *et al.*, 2004). While the animals in this study were kept on a natural light cycle, other natural environmental cues such as rainfall, temperature and humidity changes could not be replicated in our lab. The exogenous hormone therapies used in this study likely circumvented seasonal and environmental signals necessary for natural reproduction in the DGF, allowing for egg collection across a broader timeframe. Season may be a less important factor for assisted reproduction of DGFs held in breeding colonies because the species does not hibernate (Lannoo, 2005). In contrast, temperate anuran species or those that display explosive breeding strategies may be restrained to more seasonal (such as following hibernation) or environmentally cued reproductive patterns, even when hormone therapies are applied (Calatayud *et al.*, 2018).

For ovulating females, there was no significant difference between the four hormone treatments in the total number of eggs produced per female or relative fecundity, suggesting hormone treatment did not affect the number of eggs ovulated, and that any of the treatments could be used to obtain equivalent numbers of eggs. However, cleavage rate was significantly different between some groups, with Treatment C having the lowest average cleavage rate. While differences in sperm quality between males may have resulted in some variation in cleavage rates between the groups, Treatment C consistently had low cleavage rates (typically <50%) across a majority of the IVF dishes. Using cleavage rate as a proxy of egg quality, this suggests Treatment C may result in lower quality eggs. We hypothesize that the combination of hCG and GnRHa (used in both Treatments C and D) provides the necessary stimulation to induce ovulation in DGFs, but if priming doses are not utilized (as used in Treatment D), the eggs may not be fully mature when the ovulation stimulus is received. The LH-like effects of hCG, particularly when paired with a GnRH agonist, may be a more powerful ovulation stimulus for the DGF, as Treatments C and D had the lowest median number of treatment rounds (1.0) compared with the other groups. Arguably though this increased response rate is only beneficial if eggs of sufficient quality are produced. The priming doses included within Treatment D may assist with final maturation of the eggs and explain the improved cleavage rate. Continued refinement to the doses or timing of treatments, is worth exploring. Other hormones, such as progesterone or PMSG used as primers to assist in final egg maturation (Schuetz, 1971; Browne *et al.*, 2006a; Clulow *et al.*, 2012), may improve the number of ovulating females, egg numbers or cleavage rates observed in this study.

Overall, the total egg numbers and cleavage rates reported here were similar to, or improved, compared with those observed during the earlier study from our group. The preliminary Kouba *et al.* (2011) study used a protocol similar to Treatment D here (but with a fixed concentration of 500 IU hCG and 15 μg GnRHa as the ovulatory dose) and reported a total of 6169 eggs from nine females (~685 eggs per female) and an average cleavage rate of 39%. In the Kouba study, females which were not ‘primed’ in the months beforehand, failed to ovulate; whereas, the females which ovulated, only did so after receiving the hormone regimen for a second time several months later. However, the present study demonstrates that DGFs can be induced to ovulate on the first round of hormone treatment, as all four treatment groups had at least one female ovulate on the first round of treatment. Ultrasound was not used in the Kouba *et al.* (2011) study, but as we have shown here, ovarian state likely plays a key role in determining ovulation response following hormone treatment. If females were of low ovarian grade (Grades 1–2), this may have limited the success of the initial hormone treatments in the earlier study.

Variability in response rates between the studies may also be accounted for by differences in the dosages of hormones used relative to body weight. The range of body weights and SVLs for females in our study was quite varied, however these measures were not correlated with increased egg numbers or cleavage rates, and body weight was not found to be a significant factor to predict ovulation success. This is different from some other anuran species, were female body size is related to clutch size (Prado and Haddad, 2005). For DGFs, female body size may not predict clutch size, or the exogenous hormone treatments or captive setting (with consistent, adequate nutrition) may have allowed for smaller females to produce greater numbers of eggs. Importantly, given the variability in body weight, we recommend administering hormone treatments using weight-adjusted doses as performed in this study. Following this method, our study used concentrations of hCG between 400 and 900 IU and 12–27 μg of GnRHa. This range includes the fixed concentration dose used in Kouba *et al.* (2011), but given that many of the females used in that study were large (~50–60 g), a weight-adjusted dose (equal to 675–810 IU hCG) may have improved the initial response rate, as seen in this study. A weight-adjusted dose is also safer, especially for smaller frogs, as it reduces the possibility of administering overly high hormone dosages to frogs with smaller body size relative to the average.

This study characterized the timeline for egg deposition in the DGF. We consistently found that eggs could first be expressed from a female on Day 2 or 3 post-hormone administration and that eggs could not be expressed prior to Day 2. It is likely that ovulation and migration of the eggs into the oviducts occurs during this 2–3 day period following hormone treatment. Furthermore, if no eggs were expressed by Day 3 post-hormone administration, there continued to be no eggs expressed for the remainder of the 10-day monitoring period, demonstrating that the expected window for successful ovulation following hormone treatment is relative short for DGF. Determining the timeline of ovulation and egg deposition is crucial for aligning gamete collection from males for successful IVF.

The binary logistic model suggests that ultrasound grade was the most important factor to predict female ovulation. This is consistent with the observation that only females starting at Grade 3 or 4 ovulated following hormone treatment, while low-grade females (Grades 1–2) never ovulated. Performing additional hormone trials with low-grade females would be beneficial to confirm this pattern. Ovaries of the lower ultrasound grades may consist mainly of smaller, pre-vitellogenic follicle/oocyte complexes, which are thought to be gonadotropin independent (Kwon *et al.*, 1991; Jørgensen, 1992), potentially explaining why low-grade females did not ovulate and rarely showed increases in ultrasound grade following hormone therapy. In contrast, ovulating females demonstrated an increase in ultrasound grade from 3 or 4 up to a Grade 5 prior to successful egg expression. Grade 5 ovarian state was never observed naturally (without hormone intervention) between the study trials (Graham, unpublished), and was only seen following hormone treatment. For ovulating females, the initial ultrasound grade did not appear to influence the number of eggs or egg quality (cleavage rate). However, further studies using hormone therapy and ultrasound may clarify if there is a relationship between egg quantity or quality and initial ultrasound grade.

Ultrasound was a valuable tool when used alongside hormone treatments, and we recommend that the technology be incorporated into amphibian ART and breeding programs. The continued study and use of ultrasound and ovarian patterns in anurans would be valuable for advancements and efficiency in amphibian ART, which can often be plagued by low response rates to exogenous hormones. Ultrasound survey of the ovarian states of the females in a captive breeding population may help researchers determine which frogs are most likely to ovulate. Selecting only females that show medium or high oocyte development (Grades 3–4) may reduce the time and resources needed to successfully stimulate ovulation as well as reduce handling stress due to administering multiple hormone treatments. If DGF females show an ovarian state of 1 or 2, additional resting time is likely needed before hormone therapy can be successfully applied. Longer priming regiments of hormones or environmental modifications may be able to advance the maturation of these low-grade oocytes in some cases, although additional research in this area is needed. One potential downfall to ultrasound is that interpreting the images has a slight learning curve, but we found that after brief practice, the imaging patterns become relatively easy and consistent to interpret, particularly with an established grading scale. Each new species studied will likely require its own modified grading scale, as patterns can be somewhat unique between species (Schildger and Triet, 2001; Mannion, 2008).

In total, 16 925 DGF eggs were produced in this study across all hormone treatments and trial periods. While we only pursued the fertilization and development of a subset of the study eggs, we were still able to produce hundreds of tadpoles from this work. This study highlights the power of ART to aid in amphibian conservation, as large numbers of offspring can be produced for reintroduction programs. Considering the wild population of DGFs is limited to ~200 adult frogs, reintroduction programs using ART-produced tadpoles have the potential to dramatically bolster the wild population.

## Conclusions

Ultimately, all four exogenous hormone treatments tested in this study successfully induced ovulation in the DGF, but only when females were initially at a high ultrasound grade (Grade 3 or above). Treatments D and B (hCG and GnRHa following two low priming doses of hCG or metoclopramide plus GnRHa) could be used for ART in female DGF, as they resulted in the greatest number of ovulating females, and similar egg numbers and cleavage rates. Treatment D showed the highest average cleavage rate. We advise any hormone treatments be administered using body weight-adjusted dosages for safety and efficacy. Ultrasound imaging was a valuable tool for assessing the ovarian state of DGF females and may be useful to select the females most likely to ovulate following hormone treatments. We recommend that ultrasound imaging be utilized alongside hormone therapies for DGF breeding programs and could be incorporated into other amphibian reproduction programs as well. The results from this study have important implications for the conservation of the DGF, as the captive population requires assisted reproduction strategies to remain sustainable. Furthermore, hormone treatments determined in this study can be applied to captive breeding populations of DGFs to produce offspring for reintroduction programs.
